# Galectin-3 Secreted by Human Umbilical Cord Blood-Derived Mesenchymal Stem Cells Reduces Aberrant Tau Phosphorylation in an Alzheimer Disease Model

**DOI:** 10.1155/2020/8878412

**Published:** 2020-07-18

**Authors:** Hoon Lim, Dahm Lee, Wan Kyu Choi, Soo Jin Choi, Wonil Oh, Dong Hyun Kim

**Affiliations:** Biomedical Research Institute, R&D Center, MEDIPOST Co., Ltd., Gyeonggi-do, Republic of Korea

## Abstract

The formation of neurofibrillary tangles has been implicated as an important pathological marker for Alzheimer's disease (AD). Studies have revealed that the inhibition of abnormal hyperphosphorylation and aggregation of tau in the AD brain might serve as an important drug target. Using *in vitro* and *in vivo* experimental models, such as the AD mouse model (5xFAD mice), we investigated the inhibition of hyperphosphorylation of tau using the human umbilical cord blood-derived mesenchymal stem cells (hUCB-MSCs). Administration of hUCB-MSCs not only ameliorated the spatial learning and memory impairments but also mitigated the hyperphosphorylation of tau in 5xFAD mice. Furthermore, *in vivo* experiments in mice and *in vitro* ThT fluorescence assay validated galectin-3 (GAL-3) as an essential factor of hUCB-MSC. Moreover, GAL-3 was observed to be involved in the removal of aberrant forms of tau, by reducing hyperphosphorylation through decrements in the glycogen synthase kinase 3 beta (GSK-3*β*). Our results confirm that GAL-3, secreted by hUCB-MSC, regulates the abnormal accumulation of tau by protein-protein interactions. This study suggests that hUCB-MSCs mitigate hyperphosphorylation of tau through GAL-3 secretion. These findings highlight the potential role of hUCB-MSCs as a therapeutic agent for aberrant tau in AD.

## 1. Introduction

Alzheimer's disease (AD) is a progressive, irreversible disorder characterized by amyloid plaques that form as a result of amyloid-beta (A*β*) accumulation and neurofibrillary tangles comprising of pathological tau aggregates [1]. Tau is a major microtubule-associated protein (MAP), found in normal mature neurons, and its expression is developmentally regulated by alternative splicing with six different isoforms being expressed in the adult human brain [2]. Phosphorylated tau has important roles in the promotion of tubulin assembly into microtubules and the structural stabilization of microtubules [3, 4]. However, hyperphosphorylation of tau protein in the AD brains induces aggregation of filament bundles [5], which are the hallmark of AD progression [6]. Tau protein is also a major constituent of intraneuronal and glial fibrillar lesions in many neurodegenerative diseases, referred to as “tauopathies,” including AD [2]. Neurofibrillary degeneration induced by aberrantly hyperphosphorylated tau is observed during the clinical evaluation of AD, which leads to cognitive impairments [7]. In addition, pathological tau may result in microtubule dysfunction, leading to neuronal degeneration [8]. Therefore, lowering tau levels, stabilizing tau structure, or clearing hyperphosphorylated tau aggregates in the brain may be effective therapeutic strategies for AD. Notably, antitau antibody or neurotrophic compound treatment markedly reduces tau aggregation and improves cognitive functions in animal models [9]. However, a therapeutic agent with an optimal efficacy has not yet been elucidated.

Human umbilical cord blood-derived mesenchymal stem cells (hUCB-MSCs) have emerged as an important source of allogeneic MSC-based treatment [10] as they can be collected in a noninvasive manner and exhibit beneficial properties, including low immunogenicity [11], excellent tropism, and therapeutic paracrine action [12]. In particular, our previous studies demonstrated that the paracrine action of hUCB-MSCs has multifunctional therapeutic effects in AD, including antiapoptotic effects on neuronal cells [12, 13], promotion of neurogenesis [10], restoration of synaptic dysfunction [14], and A*β* peptide clearance [15, 16]. In the present study, we investigated whether hUCB-MSCs and their secreted factors can modulate the aberrant tau proteins in AD. We established the inhibitory effects of hUCB-MSCs on tau abnormalities and subsequently identified the soluble protein GAL-3 as an essential protein secreted by hUCB-MSCs. GAL-3 reduced the formation of aggregated and hyperphosphorylated tau both *in vitro* and *in vivo*. This study is the first to identify the paracrine factors secreted by hUCB-MSCs in response to tau toxicity and demonstrate that hUCB-MSC secretes GAL-3 as a crucial factor with inhibitory effects on abnormal tau in AD.

## 2. Methods

### 2.1. Preparation and Culture of hUCB-MSCs, Human Foreskin Fibroblast 68 (Hs68), and Human Embryo Kidney 293 (HEK293) Cells

Neonatal hUCB was collected from umbilical veins after obtaining informed maternal consent in accordance with the guidelines approved by the Institutional Review Board of MEDIPOST Co., Ltd. (MP-2015-6-2). All the procedures were conducted in strict compliance with the institutional guidelines and approved protocols. The procedures used for isolation, acquisition, and culture of hUCB-MSCs were as described previously [17]. hUCB-MSCs were cultured in the minimum essential medium (*α*-MEM; Gibco, Carlsbad, CA) supplemented with 10% (*v*/*v*) fetal bovine serum (Gibco, Gibco, 17504-044) and 50 mg/mL gentamicin (Gibco, 15710-064). In all the experiments, hUCB-MSCs were used at passage 6. Hs68 (CRL-1635; ATCC, Rockville, MD, USA) and HEK293 (CRL-1573; ATCC, Rockville, MD, USA) cells were used as controls and cultured under identical culture conditions.

### 2.2. Animal Experiments

5xFAD mice (B6SJL-Tg(APPSwFlLon,-PSEN1^∗^M146L^∗^L286V)6799Vas/Mmjax) were purchased from The Jackson Laboratory (Bar Harbor, ME, USA) and maintained in accordance with the laboratory guidelines. All animal experiments were approved by the Institutional Animal Care and Use Committee of MEDIPOST Co., Ltd. (MP-LAR-2016-6-2). 5xFAD mice display predominant features of AD amyloid pathology and develop cognitive dysfunction at 4 to 6 months of age [18]. In the present study, 3 *μ*L of recombinant human GAL-3 protein (1.0 *μ*g/kg) was inoculated into the bilateral hippocampi (AP: −2.54, ML: ±3.0, DV: −2.5 mm, with reference to the bregma) of 4-month-old 5xFAD mice using a sterile Hamilton syringe fitted with a 26-gauge needle (Hamilton Company, Reno, NV, USA) with a Pump 11 Elite microinfusion syringe pump (Harvard Apparatus, Holliston, MA, USA) and an infusion rate of 0.5 *μ*L/min. For the administration of hUCB-MSCs, 6-month-old 5xFAD mice were cannulated and subjected to cell transplantation using the intracerebroventricular approach [19], and hUCB-MSCs (15 *μ*L; 1 × 10^5^ cells) were administered via a cannula into the lateral ventricle (AP: −0.22, ML: 1.0, DV: −2.1 mm, with reference to the bregma) using a microinfusion syringe pump (Harvard Apparatus) at an infusion rate of 1.0 *μ*L/min. Brain tissues were homogenized in 3 mL of Dulbecco's phosphate-buffered saline (DPBS; Corning, Manassas, VA, 20109).

### 2.3. Western Blotting Analysis

Cells and tissue lysates were prepared by ultrasonication (Branson Ultrasonics, Slough, United Kingdom) in buffer containing 9.8 M urea, 2.8 M thiourea, 4% 3-((3-cholamidopropyl) dimethylammonio)-1-propanesulfonate, 130 mM dithiothreitol (DTT), 40 mM Tris-Cl (pH 8.8), and 0.1% sodium dodecyl sulfate. Protein levels were measured using Bradford assays (Bio-Rad Laboratories, Inc., Hercules, CA). For the immunoblot analysis, BOLT 4%–12% Bis-Tris gels (Life Technologies, Carlsbad, CA, USA) were electrophoretically transferred to nitrocellulose membranes. Each membrane was blocked in 5% skimmed milk and incubated with primary antibodies overnight at 4°C. After reaction with human recombinant protein-conjugated secondary antibodies at room temperature (RT) for 1.5 h, the immunoreactivity was detected using an ECL detection kit (GE Healthcare Life Sciences, Little Chalfont, UK). The antibodies used were as follows: anti-Tau (phospho T181; Abcam, Cambridge, United Kingdom), anti-Tau (phospho T231; Abcam), anti-Tau (phospho S396; Abcam), anti-Tau (phospho S404; Abcam), anti-glycogen synthase kinase 3 beta (GSK-3*β*; phospho Y216; Abcam), anti-total GSK-3*β* (Abcam), and anti-total tau (Wako, Osaka, Japan).

### 2.4. Immunoprecipitation

Extracts of total brain tissue were prepared in an immunoprecipitation buffer containing 50 mM Tris (pH, 7.8), 150 mM NaCl, 1 mM EDTA, 5 mM NaF, 1 mM Na_3_VO_4_, 1 mM Na_4_P_2_O_7_, 1.5 mM MgCl_2_, 1 mM DTT, 10% glycerol, 0.5% NP-40, and various protease inhibitors (complete, EDTA-free; Roche). The extracts were centrifuged for 10 min at 13,000 ×g at 4°C, and the supernatants were subjected to immunoprecipitation and analysis using western blotting.

### 2.5. Small Interfering RNA (siRNA) and Reverse Transcription-Polymerase Chain Reaction (RT-PCR)

siRNAs for human GAL-3, growth differentiation factor-15 (GDF-15), and cluster of differentiation (CD) 147 were purchased from Dharmacon (Lafayette, CO, USA) and transfected using DharmaFECT (Dharmacon). Total RNA was isolated using the TRIzol Reagent (Thermo Fisher Scientific Inc. Waltham, MA, USA) following the manufacturer's protocol. The SuperScript® III Reverse Transcriptase kit was used for cDNA synthesis. PCR reactions were performed using the following oligonucleotides: Human GAL-3: sense, 5′-GGC CAC TGA TTG TGC CTT AT-3′/antisense, 5′-TCT TTC CCT TCC CCA GT-3′; human GDF-15: sense, 5′-AGA TGC TCC TGG TGT TGC TG-3′/antisense, 5′-CTG GTG TTG CTG GTG CTC TC-3′; human CD147: sense, 5′-GTC CGA TGC ATC CTA CCC TCC TAT-3′/antisense, 5′-CCC GCC TGC CCC ACC ACT CA-3′; and human *β*-actin: sense, 5′-GAC CTT CAA CAC CCC AGC CA-3′/antisense, 5′-CCC AGG AAG GAA GGC TGG AA-3′.

### 2.6. Inhibition of Tau Aggregation/Tau Disaggregation Assays

Previous studies have well-established tau aggregation assays using ThT fluorescence [20]. Human tau K18 fragments (125 amino acids, 0.5 mg/mL) were incubated at 37°C without shaking in buffers(0.1 mg/mL heparin (Sigma); DTT (Sigma); and DPBS (Corning), pH 7.4) for 2, 3, and 5 days to test inhibitory effects of tau aggregation or for more than 5 days to examine tau disaggregation in the presence or absence of recombinant proteins and cells. After incubation with thioflavin T (ThT; 5 *μ*M in 50 mM glycine buffer, pH 8.9) for 3 h, the samples were plated in triplicate in a 96-well black plate with a clear bottom. ThT fluorescence was recorded at excitation wavelengths of 450 nm and emission wavelengths of 485 nm using an EnSpire Multimode Plate Reader (PerkinElmer, Waltham, Massachusetts, USA).

### 2.7. hUCB-MSC Coculture System and Recombinant Protein Treatment

Before hUCB-MSC coculture, a tau K18 fragment mixture (with heparin and DTT) was placed in multiwell plates in the presence of buffers. Then, hUCB-MSCs (2 × 10^4^ cells/cm^2^) were cocultured in the upper chamber of a Transwell device (pore size, 1 mm; BD Biosciences, Franklin Lakes, NJ, USA) in serum-free conditions. The final concentrations of recombinant human GAL-3 and GDF-15 were both 20 ng/mL and that of CD147 was 100 ng/mL.

### 2.8. Immunofluorescence

Anesthetized mice were fixed by cardiac perfusion of phosphate-buffered saline (PBS) and 4% paraformaldehyde in PBS. Mouse brains were carefully dissected, postfixed for 24 h at 4°C in the same fixative solution, and incubated in 20% sucrose at 4°C until equilibration. The fixative was discarded by aspiration, and the brains were washed with PBS. Sequential 30 *μ*m-thick coronal sections were obtained using a cryostat (CM1850UV; Leica Microsystems GmbH, Wetzlar, Germany) at 22°C. The sections were blocked in 5% normal goat serum and 5% normal horse serum (VECTOR Laboratories, Burlingame, CA, USA). The tissues were permeabilized with 0.3% Triton X-100 and immunofluorescence was performed using standard methods with the following antibodies: anti-human mitochondria (Merck Millipore, Burlington, Massachusetts, USA), anti-3R-tau (Wako), anti-Tau (phospho T181; Abcam), and anti-tau (phospho S404; Abcam). Alexa 488 and Cy3-conjugated secondary antibodies (Jackson ImmunoResearch Europe Ltd., Newmarket, United Kingdom) were used to visualize the immune complexes. Antibody labeling was visualized under an LSM 800 confocal microscope (Carl Zeiss AG, Jena, Germany).

### 2.9. AlphaLISA Binding Assays

The FRET-based indirect AlphaLISA (Amplified Luminescent Proximity Homogeneous) assay was used to determine the saturation binding. The first sandwiching polyhistidine- (His-) tagged IgG was bound to donor beads and the second sandwiching anti-GAL-3 (Abcam) was captured by anti-rabbit IgG acceptor beads. Anti-His donor beads and rabbit acceptor beads were purchased from PerkinElmer and used at a final concentration of 20 *μ*g/mL. The 1× assay buffer (0.5% in DPBS (Corning)) was distributed into each well of a 1/2 AreaPlate-96 (PerkinElmer). Before the experiments, we prepared a 5× working solution of untagged GAL-3 and His-tagged tau K18 fragments in 1× assay buffer and performed serial dilution of all the proteins in Eppendorf tubes on ice. Donor and acceptor beads were prepared from the 5× working solution (100 *μ*g/mL). For a subset of the experiments, untagged GAL-3 protein and His-tagged tau K18 fragments were added to a white 1/2 AreaPlate-96 and incubated for 60 min at 4°C with a TopSeal adhesive seal. Indirect AlphaLISA was performed following the manufacturer's protocol, which involved capturing of the untagged proteins by adding an anti-GAL-3 antibody to the plate with the proteins. In another subset of experiments, acceptor and donor beads were added to the abovementioned 96-well plates at a final concentration of 20 *μ*g/mL and incubated with a TopSeal adhesive seal for 60 min at RT in the dark. The fluorescence was measured using the EnSpire Multimode Plate Reader (PerkinElmer), and response data were exported and analyzed using Combine graphs in a layout with the GraphPad Prism (GraphPad, San Diego, USA) software. The binding of tau K18 fragments to GAL-3 was reflected by the calculated Kd values in the saturation binding assay. The Kd value is the concentration at which the binding signal reaches 50% saturation, and it is a measure of the binding affinity [21, 22]. The binding efficiency takes different saturation levels into account by calculating the ratios of the maximum binding signals and Kd values.

### 2.10. Behavioral Tests

#### 2.10.1. T-maze Test

T-maze was constructed with one start arm and two-goal arms that formed a “T” shape. Spontaneous alternation performance was tested as previously described [23]. Each mouse was placed in the center of the symmetrical T-maze and allowed to explore freely through the maze. The sequence and the total number of arms entered were recorded. The experimenters were blinded with respect to the genotype of the mice. The percentage alternation was calculated as follows: number of triads containing entries into all three arms/maximum possible alternations. The trial was started by placing the mouse into the start arm facing the goal arms. The animals were then allowed to explore freely until they were confined in the left or right goal arm. They were moved back into the start arm and allowed to move into one of the open goal arms again. All mice were subjected to two trials conducted at a 1 h interval for 2 consecutive days. Scoring was performed as follows: 0, when the same goal arm was repeatedly chosen in the same trial and 1, when different goal arms were chosen in the same trial.

### 2.11. Open Field Test

Animals were allowed to explore an empty field (44.5 × 44.5 cm) for 20 min without any disturbing factors. They were gently placed in the peripheral area of the field. Auto-Track software (version 5.00) and the Opto-Varimex-5 Auto-Track device (Columbus Instruments, OH, USA) were used to measure the patterns of agility and rearing as indicators of locomotion and exploration, respectively.

### 2.12. Human Cytokine Antibody Array

The conditioned medium was collected from tau K18 fragments and the hUCB-MSC cocultures under inducing conditions for tau aggregation. The Human Cytokine Antibody Array C11 (Raybiotech Inc., Norcross, GA) was used to detect the secreted proteins according to the manufacturer's protocol. Membranes were incubated in the blocking buffer for 30 min at RT and each growth medium overnight at 4°C. After washing, the membranes were incubated with a diluted biotinylated antibody cocktail for 2 h at RT. After the second washing step, the membranes were incubated in horseradish peroxidase-conjugated streptavidin (1 : 1000) for 2 h at RT. After the third washing step, the signals were detected using the ChemiDoc™ Imaging System (Bio-Rad Laboratories Inc.) and quantified with the ImageJ software (National Center for Biotechnology Information, National Institutes of Health, Bethesda, MD).

### 2.13. Enzyme-Linked Immunosorbent Assay (ELISA)

A human GAL-3-specific ELISA kit (R&D Systems, Inc.) was used to determine the GAL-3 levels according to the manufacturer's instructions. The results were analyzed using a VERSAmax microplate reader (Molecular Devices, Sunnyvale, CA, USA).

### 2.14. Statistical Analysis

All data are presented as mean ± standard error of the mean. Student's *t*-tests were used to analyze the between-group differences. Multiple sets of data were compared using one-way analysis of variance followed by Fisher's least significant difference post hoc tests. *p* values of < 0.05^∗^ and< 0.005^∗∗^ were considered statistically significant.

## 3. Results

### 3.1. Administration of hUCB-MSCs Ameliorates Cognitive Dysfunction in AD Mice

**Figure 1:**
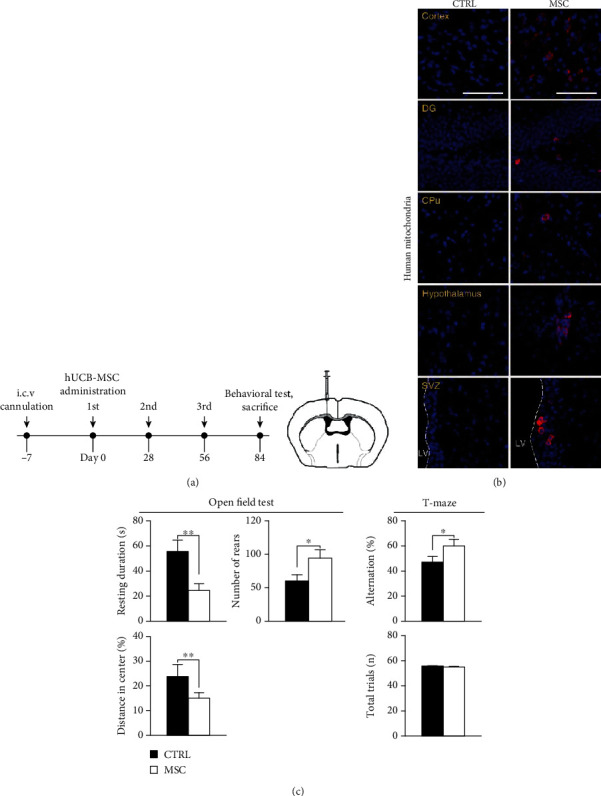
Administration of hUCB-MSCs ameliorates behavioral dysfunction in 5xFAD mice. (a) The schedule of repeated injection (3 times) of hUCB-MSCs via the lateral ventricle of 5xFAD mice, which express human APP and PSEN1 transgenes with a total of five AD-linked mutations. After 4 weeks at the last injection, mice were subjected to behavioral tests, and the brains were collected to analyze tau phosphorylation with western blotting or IF. (b) The brains injected with hUCB-MSCs were stained with antihuman mitochondria (red). Fluorescence signals were observed in the cortex, DG, CPu, hypothalamus, and SVZ (scale bar = 100 *μ*m). (c) Various behavioral tests for hUCB-MSCs-injected 5xFAD mice (open field: resting duration, the distance in center, and number of rears; T-maze: alternation; *n* = 8 per group; ^∗^*p* < 0.05, ^∗∗^*p* < 0.005). CTRL: PBS-administrated 5xFAD; MSC: hUCB-MSC-administered 5xFAD.

To determine the ameliorative effect of hUCB-MSCs on cognitive function in the AD mouse model, hUCB-MSCs were injected three times at 4-week intervals into the lateral ventricle of 6-month-old 5xFAD mice, which is the age at which these mice display cognitive dysfunction. For the control set, PBS was injected in a similar manner. Behavioral tests were conducted 4 weeks after the last injection (Figure 1(a)).

After repeated administration of hUCB-MSCs, the brains of 5xFAD mice were analyzed using immunofluorescence. Upon evaluation, we found that the transplanted hUCB-MSCs were present in the brain parenchyma (red: human mitochondria-labeled hUCB-MSCs), including the cortex, hippocampal dentate gyrus (DG), caudate-putamen (CPu), hypothalamus, and subventricular zone (SVZ) (Figure 1(b)). To evaluate the changes in the cognitive function of AD mice due to hUCB-MSC administration, behavioral tests were conducted in both the hUCB-MSC-treated 5xFAD and PBS-injected control groups. The open field test conducted to evaluate the general activity, anxiety, and exploratory behavior showed a significant improvement in locomotion (general activity, distance in the center (%), and resting duration (%)) and exploratory behavior caused by curiosity (number of rears) in the hUCB-MSC group. Moreover, a comparative analysis of changes in alternation (%) in the T-maze, used to evaluate spatial working memory, showed an ameliorative effect on the spatial memory in the hUCB-MSC group (Figure 1(c)).

These findings indicate that the administration of hUCB-MSCs can ameliorate cognitive dysfunction in the AD mouse model.

### 3.2. Administration of hUCB-MSCs Decreases Tau Phosphorylation and Inhibits the Formation of Aberrant Tau in AD Mice

**Figure 2:**
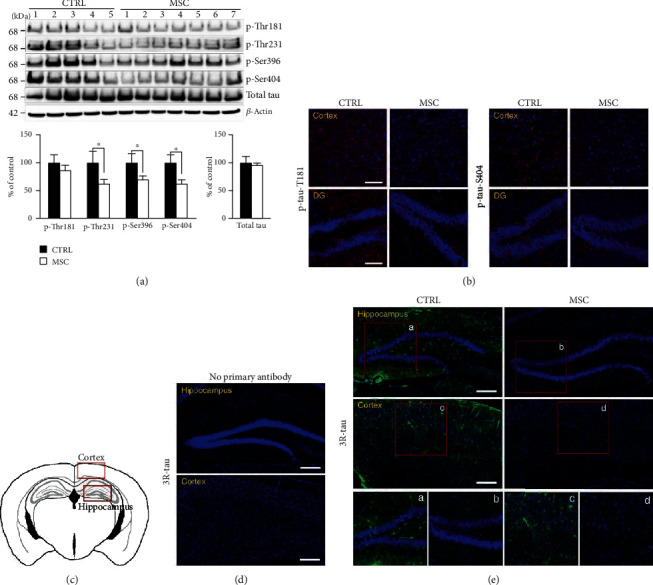
Administration of hUCB-MSCs inhibits the phosphorylation and formation of aberrant tau in 5xFAD mice. (a) Western blotting analysis showing phosphorylation and total levels of tau proteins in the right side of the whole brains in 5xFAD mice after injection of hUCB-MSCs. *β*-Actin was used as a loading control. Western blotting was analyzed using densitometric quantification (*n* = 5 for the CTRL group, *n* = 7 for the MSC group, ^∗^*p* < 0.05). (b) Sections of the cortical and DG regions in the brains were stained with specific antibodies for phosphorylated tau (red) (tyr181 or ser404) (scale bar = 100 *μ*m). (c) Coronal sections. Each red box indicates regions assessed using antibodies in (d, e). (d) Immunofluorescence performed in the absence of 3R-tau primary antibody was included to control for the nonspecific binding of the secondary antibody (no primary antibody). (e) Each tissue section was stained with DAPI and anti-3R-tau antibodies. The tile scan images acquired using a confocal microscope are shown in green (aberrant tau) in the hippocampal or cortex region (scale bar = 200 *μ*m). The boxed areas represent magnified images. CTRL: PBS-administrated 5xFAD; MSC: hUCB-MSC-administered 5xFAD.

To assess the association between the ameliorative effect of hUCB-MSCs on the cognitive function and aberrant tau pathology in AD mice, the mice were sacrificed after the behavioral tests and examined for changes in tau phosphorylation, which is significantly increased in AD and other related tauopathies. Accumulating evidence indicates that 5xFAD mice particularly develop tau hyperphosphorylation before the learning and memory impairments [24]. The brains of 5xFAD mice injected with hUCB-MSCs were examined by western blotting for identifying changes in the various disease-associated phosphorylated sites of tau, including thr181, thr231, ser396, and ser404. Administration of hUCB-MSCs significantly reduced the expression levels of these phosphorylated sites compared with that of the vehicles (Figure 2(a)). Also, immunofluorescence data revealed a decrease in the expression levels of the phosphorylated sites, including thr181 and ser404, in the hUCB-MSC group compared with the control group (Figure 2(b)).

To determine whether the inhibitory effects of hUCB-MSCs on tau phosphorylation also impacts the formation of aberrant form of phosphorylated tau in AD, the abnormal tau were examined in 5xFAD mice. Previous reports show that abnormal phosphorylation of tau triggers the aggregation of tau into filament [25] and that the microtubule-binding repeat region of tau is an important domain associated with the aggregation [26, 27]. Besides, each tau is expressed as 3-repeat (3R) and 4-repeat (4R) isoforms by alternative splicing [28]. 3R- and 4R-tau isoforms accumulate in a hyperphosphorylated state in the AD brains, and the 3R-tau contributes to the aggregation during the development of tau pathology [5, 29, 30]. Therefore, to understand the formation of the abnormal tau, we used a specific antibody for the 3R-tau isoforms targeting the microtubule repeated binding domain.

Immunofluorescence analysis showed that the 3R-tau antibody-positive signal was decreased in the hUCB-MSC group compared with the control group (Figures 2(c) and 2(e)). The no primary antibody, serving as a negative control, confirmed that this staining was not an artifact (Figure 2(d)).

Taken together, these results indicate that the administration of hUCB-MSCs not only decreases tau phosphorylation but could also inhibit the formation of aberrant tau, a stage that follows tau hyperphosphorylation in AD mice.

### 3.3. Decrease in Tau Aggregation by hUCB-MSC-Secreted GAL-3

**Figure 3:**
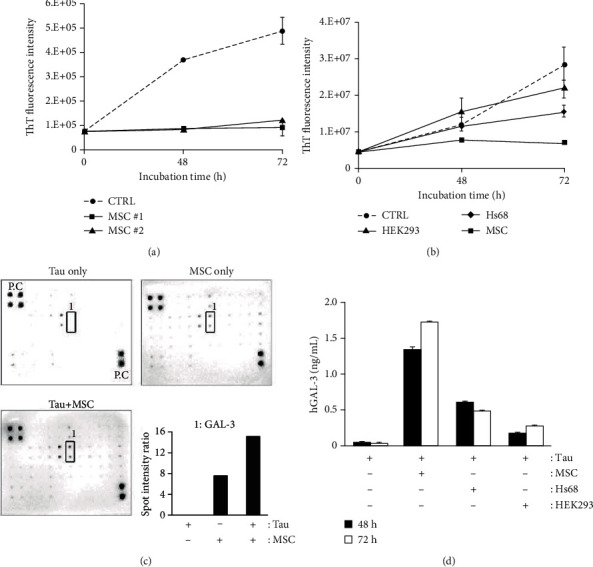
GAL-3 secreted from hUCB-MSCs suppresses aggregation of tau *in vitro*. (a, b) After treatment with two hUCB-MSC lots in tau K18 fragments under the aggregation-inducing condition, each conditioned medium was analyzed by the ThT fluorescence assay to identify the relative intensity of aggregated tau levels. Tau aggregation-inducing condition was used as a control (mean ± SEM, *n* = 3 per group). (c) Cytokine arrays were conducted with the conditioned medium. Box 1 indicates GAL-3 protein levels under each condition. A densitometric analysis of GAL-3 was performed. (d) Conditioned media from various human originated cells in the Transwell system were analyzed with ELISA to identify the relative quantity of secreted human GAL-3. (mean ± SEM, *n* = 3 per group). MSC: hUCB-MSCs; Hs68: human foreskin fibroblast; HEK293: human embryonic kidney 293.

We next determined the effect of hUCB-MSCs on tau aggregation using the ThT assay *in vitro*. Recombinant K18 fragments from the paired helical fragments (PHF) core of full-length human tau, which is an important microtubule-binding repeat domain involved in tau aggregation, were used for the experiments [27] and were mixed with heparin to induce aggregation [31]. Incubation with hUCB-MSCs dramatically inhibited tau aggregation that persisted during the incubation period (Figure 3(a)), whereas no clear inhibitory effect on tau aggregation was observed after incubation with Hs68 and HEK293 cells (Figure 3(b)).

Aggregation-induced tau K18 fragments were cocultured with hUCB-MSCs to identify specific paracrine factors secreted by hUCB-MSCs that are associated with the inhibition of tau aggregation. The cocultured medium was analyzed using a human cytokine antibody array. The GAL-3 level was significantly increased in the hUCB-MSC^+^ aggregation-induced tau K18 group compared with the aggregation-induced tau K18 only group (control). Although GAL-3 was also detected in the hUCB-MSC only group, its expression was significantly higher when hUCB-MSCs were incubated with aggregation-induced tau K18 fragments (Figure 3(c)). Using human GAL-3-specific ELISA, higher expressions of GAL-3 were observed after coculture with hUCB-MSCs (an approximately twofold increase) than after coculture with HEK293 and Hs68 cells (Figure 3(d)).

These results demonstrate that hUCB-MSCs can inhibit the formation of tau aggregates and that the paracrine effect of hUCB-MSC-secreted GAL-3 is specifically increased under conditions favoring tau aggregation.

### 3.4. Gal-3 Is Essential for the Inhibition of Tau Aggregation *In Vitro*

**Figure 4:**
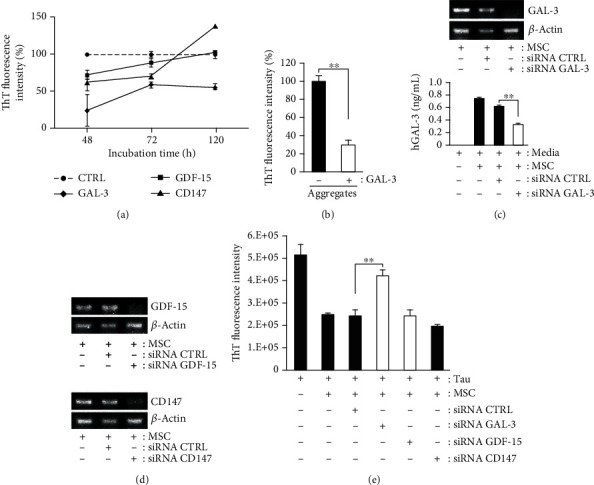
GAL-3 secreted from hUCB-MSCs is an essential factor for inhibition of tau aggregation. (a) A variety of factors were administered to the K18 fragments under the aggregation-induced condition, and each conditioned medium was used to analyze the level of aggregated tau using the ThT fluorescence assay. Tau aggregation-inducing condition was used as a control (mean ± SEM, *n* = 3 per group). (b) The ThT assay showed that the effects of GAL-3 on tau disaggregation were greatly substantial (mean ± SEM, ^∗∗^*p* < 0.005, *n* = 3 per group). (c) hUCB-MSCs were transfected with control or GAL-3-siRNA at 37°C overnight. On the following day, the cells were incubated with tau K18 fragments under the aggregation-inducing condition. GAL-3 and *β*-actin mRNA and protein levels were, respectively, assessed by RT-PCR and ELISA (mean ± SEM, ^∗∗^*p* < 0.005 versus control-siRNA-treated hUCB-MSCs). (d) siRNAs of GDF-15 or CD147 could effectively knockdown GDF-15 or CD147 expressions, as analyzed with RT-PCR. (e) Tau K18 fragments were treated with conditioned media derived from hUCB-MSCs, in which CTRL, GAL-3, GDF-15, and CD147 were knockdown by siRNA. The relative quantity of aggregated tau levels was estimated by the ThT fluorescence assay (mean ± SEM, ^∗∗^*p* < 0.005 versus control-siRNA-treated hUCB-MSCs).

To confirm the role of GAL-3, a recombinant human GAL-3 (rhGAL-3) protein was incubated with aggregation-induced tau K18 fragments, and the medium was analyzed by the ThT fluorescence assay. During incubation, treatment with rhGAL-3 protein (20 ng/mL) inhibited aggregation; however, none of the other secreted proteins from hUCB-MSCs (Supplementary Figure 1), such as GDF-15 (20 ng/mL) or CD147 (100 ng/mL), were able to inhibit the aggregation-induced tau K18 fragments (Figure 4(a)). Treatment of tau K18 aggregates with rhGAL-3 protein was able to significantly reduce the ThT fluorescence intensity (Figure 4(b)).

In addition, the treatment of hUCB-MSCs with GAL-3-specific siRNA markedly reduced the mRNA and protein levels of GAL-3 (Figure 4(c)). Next, GAL-3-deficient culture media harvested from GAL-3 knockdown hUCB-MSCs, upon treatment with aggregation-induced tau K18 fragments, showed reduced inhibition of tau aggregation as compared to that in the control group (scrambled siRNA-transfected hUCB-MSCs; Figure 4(e)). However, no change was observed in the level of tau aggregation, after treatment with hUSB-MSC cell cultures incubated with GDF-15- and CD147-specific siRNAs or the control group, confirming the specificity of GAL-3 in tau aggregation. (Figures 4(d) and 4(e)).

These results indicate that hUCB-MSC-secreted GAL-3 is an essential factor for specific inhibition of tau aggregation in an *in vitro* assay.

### 3.5. GAL-3 Decreases Tau Phosphorylation by Modulation of GSK-3*β* in AD Mice

**Figure 5:**
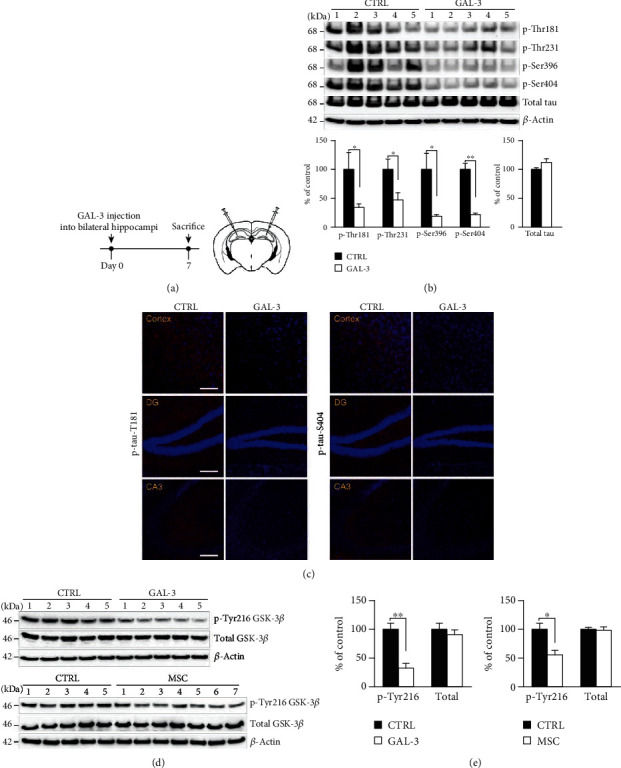
GAL-3 attenuates tau pathology in 5xFAD mice. (a) Schematics of human recombinant GAL-3 injection into the hippocampi of 5xFAD mice and the schedule of animal sacrifice. (b) After 1 week, GAL-3-injected 5xFAD mouse brains were extracted and assessed by western blotting with various phosphorylated tau antibodies. *β*-Actin was used as a loading control. Western blotting was analyzed using densitometric quantification (*n* = 5 per group, ^∗^*p* < 0.05, ^∗∗^*p* < 0.005). (c) Sections of the cortical and DG regions were stained with specific antibodies for phosphorylated tau (red), tyr181, or ser404 (scale bar = 100 *μ*m). (d) GAL-3-injected or hUCB-MSC-administrated 5xFAD mouse brains were analyzed by western blotting with tyrosine 216 phosphorylated GSK-3*β* or total GSK-3*β* antibodies. *β*-Actin was used as a loading control. (e) Densitometric quantification of western blotting in (d) (^∗^*p* < 0.05, ^∗∗^*p* < 0.005, *n* = 5 per group or *n* = 5 ~ 7 per group). CTRL: PBS-administrated 5xFAD; GAL-3: GAL-3-injected 5xFAD; MSC: hUCB-MSC-administrated 5xFAD; DG: dentate gyrus; CA3: cornu ammonis 3.

To evaluate the effects of GAL-3 on tau pathology in AD mice, rhGAL-3 protein (1.0 *μ*g/kg) was injected into the bilateral hippocampi of 4-month-old 5xFAD mice. Mice were then sacrificed after 7 days (Figure 5(a)) to study the expression of various disease-associated phosphorylated sites of tau, including thr181, thr231, ser396, and ser404. The expression of these sites was significantly reduced in the rhGAL-3 protein group compared with the PBS-injected control group (Figure 5(b)). Results from immunofluorescence also showed a reduction in the expression of phosphorylated sites, including thr181 and ser404, after rhGAL-3 protein administration (Figure 5(c)).

To understand the mechanism underlying the modulation of tau phosphorylation, we investigated the role of kinases located upstream of tau. In particular, we tried to establish the association between GAL-3 and GSK-3*β*, a known tau phosphorylation-regulating kinase in AD, *in vivo*. Using western blotting, we showed that GSK-3*β*-tyr216 phosphorylation was significantly reduced after the administration of rhGAL-3 protein (75.6% decrease relative to that in the control group) and GAL-3-secreting hUCB-MSCs (50.9% decrease relative to that in the control group) in 5xFAD mouse brains (Figures 5(d) and 5(e)).

These findings suggest that GAL-3 modulates tau phosphorylation by reducing phosphorylation at the tyr216 residue of activated GSK-3*β* in AD mice.

### 3.6. GAL-3 Prevents the Formation of Aberrant Tau and Directly Interacts with Tau in AD Mice

**Figure 6:**
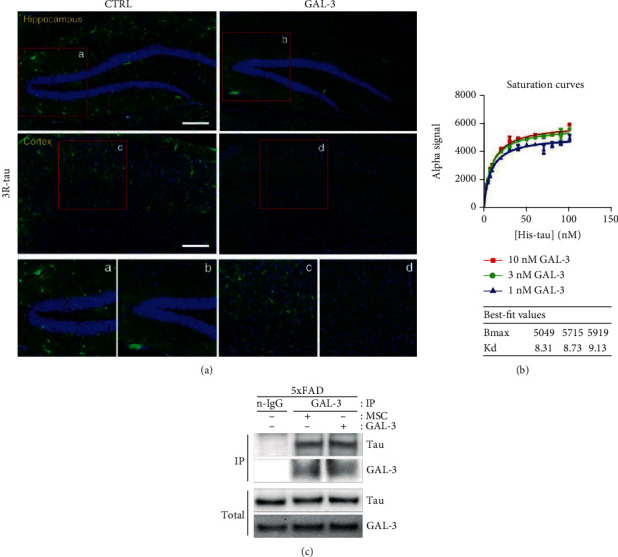
GAL-3 alleviates the formation of aberrant tau through protein binding in 5xFAD mice. (a) Each tissue section was stained with DAPI and anti-3R-tau antibodies. The tile scan images using a confocal microscope are shown in green (aberrant tau) in the hippocampal or cortical region (scale bars = 200 *μ*m). The boxed areas represent magnified images. (b) Saturation curves were determined by fluorescence intensities of the interaction between tau-K18-His fragment and GAL-3 in AlphaLISA format by the FRET-based assay. Tau-K18-His fragment and GAL-3 proteins were incubated together at a wide range of concentrations (Tau-K18-His fragment: 0 ~ 100 nM, GAL-3: 1 ~ 10 nM) followed by analysis with specific Alpha Donor/Acceptor beads. (c) hUCB-MSC- or GAL-3-injected 5xFAD mouse brains were extracted, and anti-GAL-3 immunoprecipitates and total lysates were analyzed by western blotting with anti-Tau or anti-GAL-3 antibodies. The n-IgG used as a control was immunoprecipitated by normal IgG antibody. CTRL: PBS-administrated 5xFAD; GAL-3: GAL-3-injected 5xFAD; MSC: hUCB-MSC-administrated 5xFAD.

To confirm the role of GAL-3 secreted from hUCB-MSCs in the aberrant formation of phosphorylated tau in AD, we evaluated the changes in abnormal tau after rhGAL-3 protein injection in AD mice. Immunofluorescence using the 3R-tau antibody for the 3-repeat tau isoform revealed that the 3R-tau-positive signal was decreased in the GAL-3-treated group compared with that of the control group (Figure 6(a)).

In addition, to verify the possibility of interference in aberrant tau by protein-protein interaction, we measured the binding kinetics between the tau K18 fragments and GAL-3 using the fluorescence resonance energy transfer- (FRET-) based AlphaLISA assay. The binding ability between the two proteins was reflected by Kd values calculated using the AlphaLISA saturation binding assay. In this experiment, tau K18 fragments were used as an analyte at a concentration ranging from 0 to 100 nM. The assay demonstrated that the binding of tau K18 fragments to GAL-3 (1, 3, and 10 nM) was saturable, with a high binding affinity. The Kd values at 1, 3, and 10 nM were 8.31, 8.73, and 9.13 nM, respectively (Figure 6(b)). This result indicates a high degree of direct interaction between the tau K18 fragments and GAL-3.

Moreover, we analyzed the brain extract of AD mice using immunoprecipitation in order to determine the actual binding between tau and GAL-3 *in vivo*. After immunoprecipitation using the GAL-3 antibody, the binding complex of GAL-3-tau was selected for immunoblotting, and the binding was confirmed by the tau and GAL-3 expressions. The expressions of both GAL-3 and tau were detected in the brain extract using the GAL-3 antibody, but not with the negative IgG antibody (n-IgG) (Figure 6(c)).

These results suggest that hUCB-MSC-secreted GAL-3 can hinder the formation of abnormal tau by directly binding with tau in AD mice.

## 4. Discussion

In this study, we demonstrated the inhibitory effect of hUCB-MSCs on the formation of aberrant tau and subsequently identified a soluble protein GAL-3 as an essential protein secreted by hUCB-MSC in response to tau abnormalities. In addition, we showed that the interaction between GAL-3 and tau reduced the formation of abnormal tau by directly binding and inhibiting the hyperphosphorylation of tau. Moreover, we determined the mechanism underlying the inhibition of tau hyperphosphorylation by GAL-3.

Abnormal hyperphosphorylation of tau protein and the development of tau tangles along with amyloid accumulation are the fundamental characteristics of AD progression in the human brain. Induction of structural stabilization and clearing of hyperphosphorylated tau aggregates by tau modulation have emerged as alternative therapies [32, 33]. Tau protein was initially identified as a microtubule-associated protein in the brain microtubules [34]. Structurally, it is divided into the acidic region of the N-terminal portion, a proline-rich region, microtubule-binding four-repeat domains (RD1~4), and the C-terminal region, and the alternative splicing of the tau primary transcript in the central nervous system produces the six isoforms of 352-441 amino acids [35]. Specifically, the exon 10 (RD2) contains the microtubule-binding region and the insertion of exon 10 leads to the 4-repeat (4R) tau isoforms whereas the 3-repeat (3R) tau isoforms are produced without the exon 10 [28]. 3R- and 4R-tau contribute to the abnormal accumulation in tau pathology in AD brain [36]. In particular, aberrant modifications of hyperphosphorylated tau from increased *β*-structure levels in the repeat domains eventually lead to the formation of PHFs in AD [37]. Such modifications of tau in the intracellular or extracellular space may be toxic to neurons [38]. Therefore, inhibition of abnormal hyperphosphorylation and tau aggregation has become a strategically important therapeutic target. However, despite extensive efforts for drug development, an approved drug has not yet been developed [39].

Studies have revealed that hUCB-MSCs exert a therapeutic effect on AD via the secretion of therapeutic factors. The effects of hUCB-MSCs, including amyloid clearance, anti-inflammatory effects, and recovery of synaptic function indicate that hUCB-MSCs have the capacity to improve the overall environment in AD, which has various pathologies [14, 15, 40, 41]. Therefore, hUCB-MSCs might have multifunctional therapeutic effects and play a pivotal role as a therapeutic agent for AD. In the present study, we presented clear evidence supporting a new therapeutic role of hUCB-MSCs in AD: modulation and inhibition of the abnormal hyperphosphorylation of tau. Moreover, we also demonstrated the modulatory effect of hUCB-MSCs on tau through a paracrine action in an AD environment and identified GAL-3 as the primary associated paracrine factor.

GAL-3, a member of the *β*-galactoside-binding protein family, is a multifunctional protein involved in both intracellular and extracellular functions [42, 43]. It is an extracellular space protein secreted by a nonclassical secretory pathway and it not only modulates the basic cellular functions, including cell-cell interactions, proliferation, and differentiation, but also plays a role in the pathogenesis of many human diseases and promotion of neural cell adhesion and neurite outgrowth [44–46]. GAL-3 plays a critical role in MSC survival, migration, and therapeutic application [47]. In our previous study, we demonstrated the antiapoptotic role of hUCB-MSC-secreted GAL-3 in neuronal cells [13]. GAL-3 aids in autophagy-mediated removal of ruptured phagosomes and lysosomes via recognition molecules [48], and galectin-8-mediated selective autophagy prevents seeded tau aggregation [49]. In contrast, GAL-3 enhances A*β* oligomerization and A*β* toxicity, while GAL-3 deletion decreases the microglia-associated immune response and improves cognitive behavior in AD mice [50, 51]. These conflicting functional roles of GAL-3 could be due to the difference in cell types expressing GAL-3 or due to the difference in response to the stimulating environment. In the present study, we demonstrated that GAL-3 had a modulating effect on tau protein phosphorylation and an inhibitory potential on the formation of aberrant tau.

Tau protein functions in the brain via kinase-regulated phosphorylation [52]. Of these kinases, GSK has two closely related isoforms, GSK-3 alpha and GSK-3*β*. The latter is a proline-directed kinase that plays a key role in controlling the numerous signaling pathways in the central nervous system and modulates important cellular processes [53, 54]. GSK-3*β* is structurally activated by autophosphorylation at the Tyr216 residue and inactivated by phosphorylation at the Ser9 residue [55]. Activation of GSK-3*β* results in hyperphosphorylation of most sites of tau and increases tau phosphorylation in AD [56–58]. Moreover, GSK-3*β* directly participates in the microtubule destabilization and PHF formation in the AD brains [59, 60]. Recently identified GSK-3*β*-phosphorylated sites of tau include the Thr181, Ser199, Thr231, Ser396, Ser404, Ser413, Ser46, Thr50, and Ser202/Thr205 [54, 61]. In the present study, we show that GAL-3 modulates the expression of GSK-3*β*. The present findings are of clinical significance because the hUCB-MSC-secreted protein GAL-3 may reduce the tau phosphorylation through GSK-3*β* mediation and interfere with AD progression due to the aberrant tau pathology.

In the present study, we selected 5xFAD as the double-transgenic AD mouse model of choice as these mice mimic most of the pathologic alterations, including early accumulation of A*β*, neuronal loss, and cognitive deficits similar to patients with AD [62]. Although these double-transgenic AD mouse models, with only APP and PS1 mutation, are not exact replicable models of tauopathy, they have the potential to exhibit hyperphosphorylated tau as punctuate deposits and neurofibrillary changes [62, 63]. Further, A*β* peptides can induce the formation of tau fibrils in culture and stimulate tau hyperphosphorylation in AD model [30, 64, 65]. These previous reports show that the double-transgenic AD mouse model may be partially helpful in understanding the aberrant tau formation. In this study, we present the possible regulation of abnormal tau by hUCB-MSCs in an AD model.

Using AlphaLISA binding assay and immunoprecipitation analysis of the brain extract from 5xFAD mice, we demonstrated binding between tau and GAL-3. Binding between proteins has been known to inhibit aggregation, for example, small molecule curcumin binds to the monomeric form of *α*-synuclein, which belongs to aggregation-prone proteins, and prevents *α*-synuclein aggregation [66]. Thus, based on the results from this study we propose that hUCB-MSC-secreted GAL-3 protein could inhibit the aggregated formation of tau by its interaction with tau.

Further studies are needed to investigate the pathways associated with kinases that function as tau modulators. In order to verify the ameliorative effect of hUCB-MSCs on abnormal tau, it is important to establish an *in vitro* model, which allows the coculture of tau with hUCB-MSCs, thus unraveling the mechanism behind the beneficial effects of GAL-3 secreted by hUCB-MSCs. Moreover, validation of the efficacy of hUCB-MSC-secreted GAL-3 in tauopathy animal models, such as Tau P301S Tg mice, will further demonstrate its applicability as a therapeutic agent for neurodegenerative diseases associated with tauopathy.

## 5. Conclusion

This study suggests that hUCB-MSCs modulate hyperphosphorylated tau *in vivo* and aggregation of tau *in vitro*, which are the major pathological hallmarks of AD. hUCB-MSC-secreted GAL-3 can inhibit tau phosphorylation by modulating GSK-3*β* and suppressing the formation of aberrant tau by interacting with the tau protein. These findings indicate that abnormal tau in AD may be modulated by the paracrine action of proteins secreted by hUCB-MSCs, and GAL-3 may contribute to the beneficial effects of hUCB-MSCs. Our findings (tau protein modulation by hUCB-MSCs) present a new direction and possibility for the role of stem cell therapy in AD.

## Data Availability

All the data used to support the findings of this study are available from the corresponding author upon reasonable request.

## Supplementary Materials

Supplementary Materials Supplementary Figure 1: analysis of human cytokines secreted from hUCB-MSCs in conditioned media.
