# Supplementary material for: Galectin-3 Secreted by Human Umbilical Cord Blood-Derived Mesenchymal Stem Cells Reduces Aberrant Tau Phosphorylation in an Alzheimer Disease Model
Source: Stem Cells Int. 2020 Jul 18;2020:8878412. doi: 10.1155/2020/8878412 (PMC7383310; doi:10.1155/2020/8878412)
Supplement: Supplementary Materials — Supplementary Figure 1: analysis of human cytokines secreted from hUCB-MSCs in conditioned media. [file 8878412.f1.docx]

**Galectin-3 Secreted by Human Umbilical Cord Blood-derived Mesenchymal Stem Cells Reduces** **Aberrant Tau Phosphorylation in an Alzheimer’s Disease Model**

Hoon Lim^1^**^§^**, Dahm Lee^1^, Wan Kyu Choi^1^, Soo Jin Choi^1^, Wonil Oh^1^, Dong Hyun Kim^1*^

*^1^Biomedical Research Institute, R&D Center, MEDIPOST Co., Ltd, Gyeonggi-do, Republic of Korea*

**^§^**This author is the main contributor to the study.

Running head: **Galectin-3 Reduces** **Aberrant Tau Phosphorylation**

Correspondence should be addressed to**:** Dong Hyun Kim, Ph.D.

Biomedical Research Institute, MEDIPOST Co., Ltd,

Gyeonggi-do 13494, Republic of Korea

Phone: +82-2-3465-6787

Fax: +82-2-3465-6754

E-mail: pooh1994@medi-post.co.kr


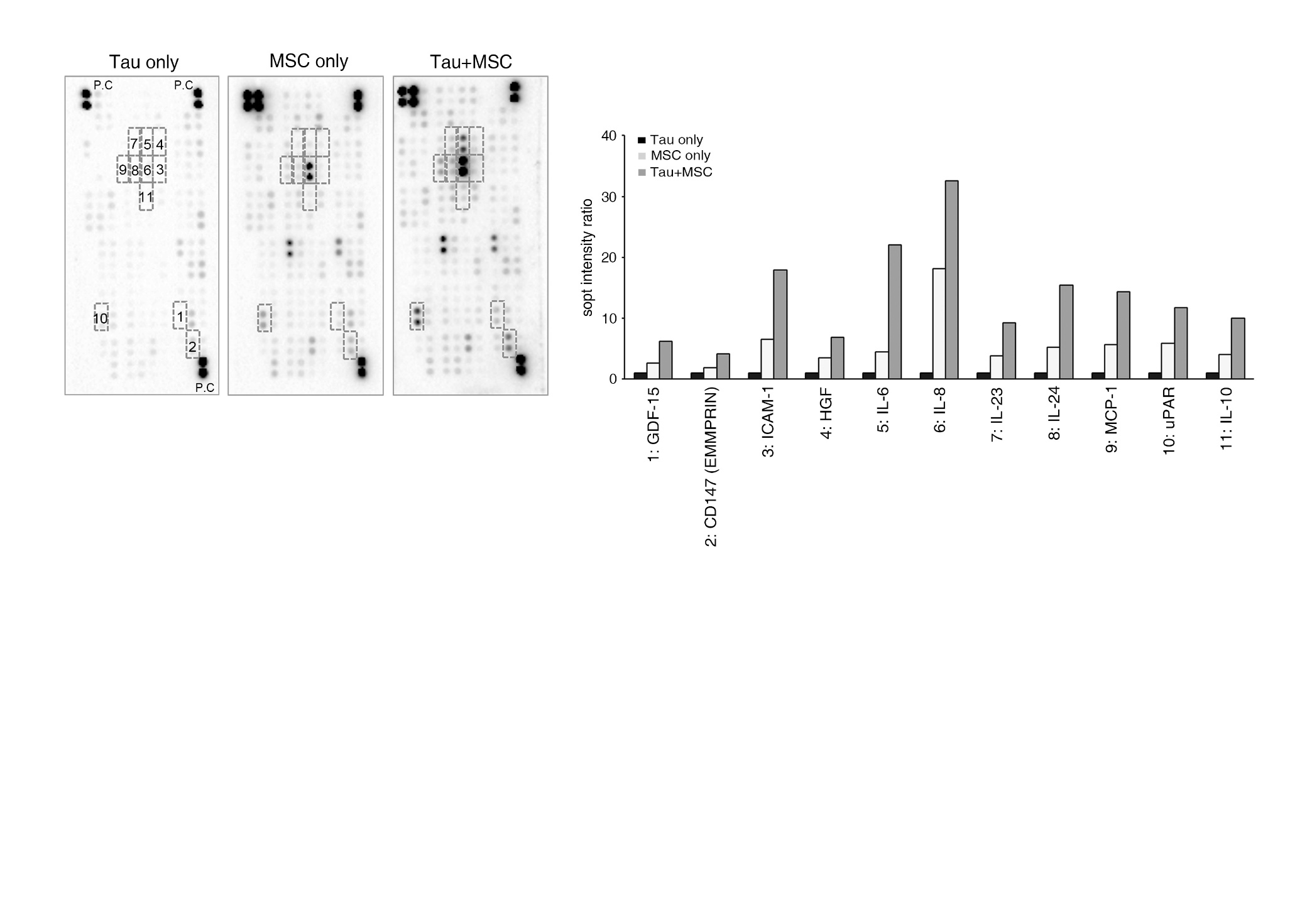


**Supplementary Figure 1.** **Analysis of human cytokines secreted from hUCB-MSCs in conditioned media.** The co-cultured medium was analyzed using a human cytokine antibody array (R&D Systems, Proteome Profiler Human XL Cytokine Array). Box indicates the expression levels of respective protein under each condition. Bar graph represents expressed protein levels determined using densitometric analysis. These proteins were remarkably increased in the hUCB-MSCs^+^ aggregation-induced tau K18 group compared with the aggregation-induced tau K18 only group (control).
